# The Application Value of Early Amplitude-Integrated Electroencephalogram in a Newborn with Nonketotic Hyperglycinemia: A Rare Case Report

**DOI:** 10.1055/a-2595-5740

**Published:** 2025-05-27

**Authors:** Enting Ma, Wei Li, Hong Gao, Shifeng Ma, Qianwen Chai, Li Wei, Jing Wang

**Affiliations:** 1 Department of Neonatology, Tianjin Medical University General Hospital, Tianjin, People's Republic of China; 2 Department of Pediatric Ward, Tianjin Medical University General Hospital, Tianjin, People's Republic of China; 3 Department of Nursing, Tianjin Medical University General Hospital Airport Hospital, Tianjin, People's Republic of China

**Keywords:** amplitude-integrated electroencephalogram, nonketotic hyperglycinemia, newborn, burst–suppression

## Abstract

**Objective:**

This study aimed to evaluate the application value of amplitude-integrated electroencephalogram (aEEG) findings in a newborn with nonketotic hyperglycinemia (NKH).

**Study Design:**

The clinical data of a neonatal patient with NKH were retrospectively analyzed. In this study, aEEG was first used to assess brain function in NKH due to *AMT* gene mutations in the Chinese mainland so far. The aEEG assessment was stratified according to its background pattern, sleep–wake cycle (SWC), and seizure activity, which gave more objective and systemic results.

**Results:**

Seizures and burst–suppression pattern were detected on the aEEG. The background belonged to discontinuous voltage, and showed discontinuity of cerebral activity in the form of the burst–suppression pattern. The classification of SWC in this record belonged to the “No SWC” category, which meant the child had severe brain damage. A typical neonatal single seizure was found. The seizure activity lasted approximately 30 seconds. However, clinical symptoms were not observed.

**Conclusion:**

Patients with NKH often exhibit complicated clinical phenotypes, and there is a lack of specific symptoms, especially the symptoms of encephalopathy are atypical. aEEG is helpful for the early diagnosis and treatment of seizures. It can help the doctor to carry out appropriate treatment in time. The application value of aEEG in patients with NKH was significant.

Nonketotic hyperglycinemia (NKH; OMIM 605899) is a rare, congenital, and genetic metabolic disease with an estimated prevalence of 1:76,000. 1 It is an autosomal recessive disorder of glycine metabolism resulting in an excessive accumulation of glycine in all body tissues, especially in the central nervous system. 2 3 Mutations mostly occur in the *GLDC* and *AMT* genes. 1 There are only a few cases reported in the Chinese mainland so far, and the incidence is still unknown. Furthermore, most cases of NHK are caused by *GLDC* gene defects in the Chinese mainland. 4 5 6 7 In this study, a Chinese male infant diagnosed with NKH due to *AMT* gene mutations was the second case reported in the Chinese mainland.

Metabolic disorders are not symptomatic immediately after birth, and they can even manifest slowly with progressive encephalopathy. 8 Thus, accurate diagnostic evaluation of neural function in newborns with a possible metabolic disorder is very important. 9 Early recording of high-risk infants is helpful for the early diagnosis and treatment of seizures.

Amplitude-integrated electroencephalogram (aEEG) has been widely used as a continuous brain-function monitoring tool in neonatal intensive care units (NICUs). 10 11 12 The aEEG has a lot of practical advantages, such as a first-line bedside tool in patients with encephalopathy and/or seizures for longer time periods. Also, the aEEG only uses two channels rather than the multichannel array of a conventional electroencephalogram (EEG). The main features of the aEEG are background activity, presence or absence of sleep–wake cycle (SWC), and specific patterns indicating seizures. 13 aEEG has been used for more than a decade in the evaluation of infants with encephalopathy, there has been no study on the efficacy of aEEG to evaluate neonates with NKH in China.

In this study, aEEG was first used in NKH due to *AMT* gene mutations in the Chinese mainland. The aEEG assessment was stratified according to its background pattern, SWC, and the presence of electrographic seizures, which gave more objective and systemic results.

## Case Report

A 2-day-old male child of the Chinese Han population was born at full term and delivered by cesarean section, with a birth weight of 3.785 kg. The Apgar scores were 9 points at 1 minute and 10 points at 5 minutes, and did not present asphyxia. The patient's parents and sister were all healthy. There was no death of any infant or miscarriage in his family history. The neonate was lethargic since birth with poor feeding and gross hypotonia. Several days later, the child presented a deterioration of consciousness until coma. The respiratory drive developed insufficiently, which required intubation and mechanical ventilation. Blood amino acids showed elevated serum glycine (1,470 μmol/L; normal range 131–368 μmol/L). Urine gas chromatography–mass spectrometry showed increased glycine in urine. Cerebrospinal fluid (CSF) glycine was elevated (215 μmoL/L; normal range 3–10 μmol/L), and the CSF/serum glycine ratio was 0.146 (normal ≤0.02).

At last, considering the lack of effective treatment and poor prognosis of the disease, the patient's parents decided to abandon treatment. The child died on the 21st day after birth.

### Amplitude-Integrated Electroencephalogram Recording and Interpretation

The aEEG was recorded by a trained nurse in charge of the infant. The aEEG was recorded as a two-channel EEG using gold caps. Skin was cleaned with alcohol and covered with electrode jelly. Electrodes were placed on the scalp corresponding to the positions C3, P3, C4, and P4 following the International 10–20 System. According to the International 10–20 System, the reference electrode Cz and grounding electrode Fz were also placed on the scalp. The upper limit of tolerated impedance was 20 kΩ. Frequencies lower than 2 Hz or higher than 15 Hz were filtered. The raw EEG data were processed and time-compressed, then displayed on a semilogarithmic scale at a speed of 6 cm/h. During the aEEG recording, video monitoring was also recorded as soon as possible. The quality of the recordings was monitored by continuous impedance tracing and the absence of interference from other electrical devices. The duration of the recordings was at least 4 hours. Handling or routine nursing care periods were marked on the tracing. In addition, the patient's diagnoses and the relevant clinical information were also recorded. The aEEG traces were examined by two investigators. The raw EEG data were also inspected to verify the aEEG assessment. Disagreement was resolved by consulting a third investigator.

**Fig. 1:**
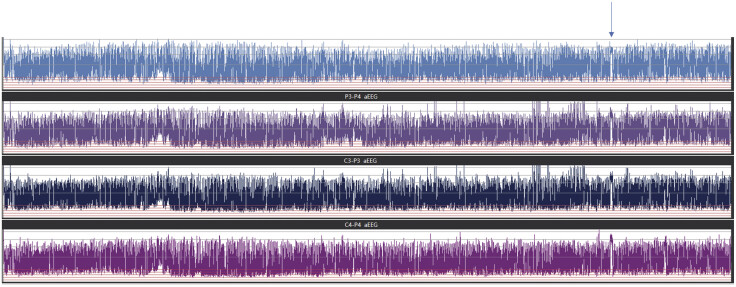
Distribution of aEEG background pattern, seizures (indicated by the arrow), and burst–suppression pattern were detected on his EEG, and no cyclic variation of the aEEG background. aEEG, amplitude-integrated electroencephalogram; EEG, electroencephalogram.

**Fig. 2:**
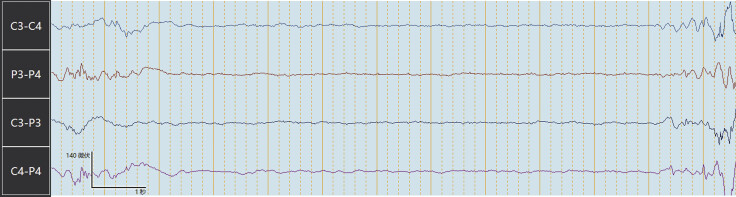
An electroencephalogram showing burst–suppression.

In this aEEG recording, the interburst interval varied with a maximum of up to 30 seconds. Seizures and burst–suppression pattern were detected on his EEG, and no cyclic variation of the aEEG background was found ( Fig. 1 ). Seizures were recognized on his EEG, but clinical symptoms were not observed. EEG showed burst–suppression ( Fig. 2 ).

### Classification of Amplitude-Integrated Electroencephalogram Background Pattern

According to the criteria described by Hellström-Westas and Rosen, 14 the aEEG background activity include five patterns: (1.) Continuous normal voltage: continuous activity with lower (minimum) amplitude around >5 μV and maximum amplitude >10 μV; (2.) discontinuous voltage (DC): discontinuous background with minimum amplitude variable, but mainly below 5 μV, and maximum amplitude over 10 μV; (3.) burst–suppression: discontinuous background with minimum amplitude without variability at 0–1[2] μV, and bursts with amplitude >25 μV; (4.) continuous low voltage: continuous background pattern of very low voltage (around or below 5 μV); (5.) inactive, flat trace: mainly inactive (isoelectric tracing) background below 3 to 5 μV.

In this study, the background belonged to DC ( Fig. 1 ), and showed discontinuity of cerebral activity in the form of a burst–suppression pattern ( Fig. 2 ). For a normal full-term neonate, the part of the raw EEG should display a continuous background rhythm in quiet sleep stage. But in this study, EEG typically showed a burst–suppression pattern. Interburst interval varied with a maximum of up to 9 seconds. The child had a signiﬁcantly longer interburst intervals (IBI) than normal full-term neonates, with a maximum of up to 6 seconds, which suggested that the child may have brain injury. 15 Longer IBI than the corresponding gestational age suggested that the child might have brain injury. The IBI period is the time of voltage suppression between two bursts, reflecting the maturity of the brain and the severity of brain injury. The electrocortical background activity with very low-voltage IBI, is associated with adverse outcome in infants. The longer the IBI is prolonged, the more serious the injury to the brain. 16 The IBI measured by EEG is directly related to developmental neurobiology, which is considered important for brain wiring during development. 17 The electrocortical background activity with very low-voltage IBI is associated with adverse outcomes in infants. 16

### Classification of Sleep–Wake Cycle

SWC in the aEEG is characterized by smooth sinusoidal variations, mainly in the minimum amplitude. The broader bandwidth represents discontinuous background activity during quiet sleep (trace alternant EEG in term infants), and the narrower bandwidth corresponds to more continuous activity during wakefulness and active sleep. Classification of SWC is as listed. (1.) No SWC: no cyclic variation of the aEEG background. (2.) Imminent/Immature SWC: some, but not fully developed, cyclic variation of the lower amplitude, but not developed as compared with normative gestational age representative data. (3.) Developed SWC: clearly identifiable sinusoidal variations between discontinuous and more continuous background activity with a cycle duration of 20 minutes. 14

SWC is defined as cyclic variations of aEEG background activity. 14 Electrophysiological maturity is intimately connected to the development of SWC. 18 The classification of SWC in this record belongs to “No SWC” ( Fig. 1 ), which means the child had severe brain damage. 15

### Seizures

Seizure activity is deﬁned as an abrupt rise of the lower border, usually accompanied by a simultaneous rise of the upper border of the recorded strip, with a simultaneous raw EEG seizure activity of at least 10 seconds duration. 14 18 19 In the compressed aEEG trend, a typical neonatal single seizure looks like a “hump” that interrupts the background trace. Epileptic seizures with a duration of <30 seconds can be difﬁcult to detect in the compressed aEEG trend. 20 Therefore, epileptic seizures need to be detected in the raw EEG.

**Fig. 3:**
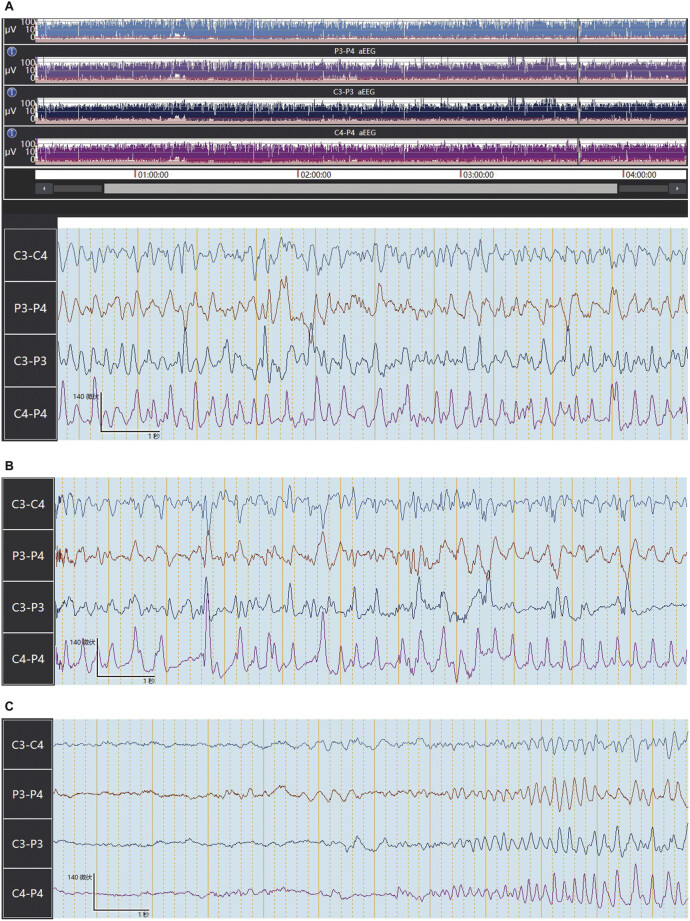
A typical neonatal single seizure was found in ( **A** ). Epileptic seizure was recorded at 3 hours and 45 minutes. The seizure activity lasted approximately 30 seconds ( **A–C** ).

In this study, a typical neonatal single seizure was found in Fig. 3 . The timing and duration of seizures were noted. Epileptic seizure was recorded at 3 hours and 45 minutes. The seizure activity lasted approximately 30 seconds ( Fig. 3A–C ). However, clinical symptoms were not observed. The presentation of the seizure was subclinical. Because of the paucity of clinical correlates, clinical diagnosis of neonatal seizures is difficult. Therefore, it was diﬃcult to identify seizures without aEEG monitoring. Early recording of NKH would be helpful for the early diagnosis and treatment of seizures, and the phenobarbital sodium was initiated immediately. The early detection and intervention for seizures may improve outcomes in patients with NKH.

## Discussion

NKH is a rare inherited genetic metabolic disease with variable clinical manifestations, which is mainly divided into classic and non-classical types. 21 The classic type is more common (84%), with progressive encephalopathy manifestations, low response, lethargy, hypotonia, myoclonic epilepsy, and symptoms such as hiccups or apneas. The symptoms worsen in a short period of time, and most children need ventilator support. 2 5 22 23 Being a fatal disease, the majority of patients die within the first week of life, and those who survive suffer from neurologic sequelae. 24 In this study, the patient belonged to a neonatal type of NKH, which might have a severe prognosis. At last, his parents decided to abandon treatment. The patient was hospitalized for 20 days in our department and died 1 day after discharge.

Patients with NKH often exhibit complicated clinical phenotypes and a lack of specific symptoms, especially the symptoms of encephalopathy are atypical. The aEEG has been used for newborns with associated risks of neurological disorders. 25 26 It has also been used for monitoring seizures and the evaluation of antiepileptic drug treatment. 27 Transient phases of clinical stabilization and normalized plasma biochemical results may not reflect the actual encephalopathic process. Serial EEGs are helpful to assess the efficacy of treatment and to modify the therapeutic approach as early as possible. 2 In this study, the aEEG was performed as early as possible in our study. There has been no study on the efficacy of aEEG to evaluate neonates with NKH in China. Clinically, the symptoms of seizures were not observed. If aEEG was not performed as early as possible, the diagnosis and treatment may be delayed. aEEG is a tool, not a treatment, but aEEG was helpful for the early diagnosis and treatment of seizures. It can help the doctor to carry out appropriate treatment at the earliest. The early detection and intervention for seizures may improve outcomes in patients with NKH.

**Fig. 4:**
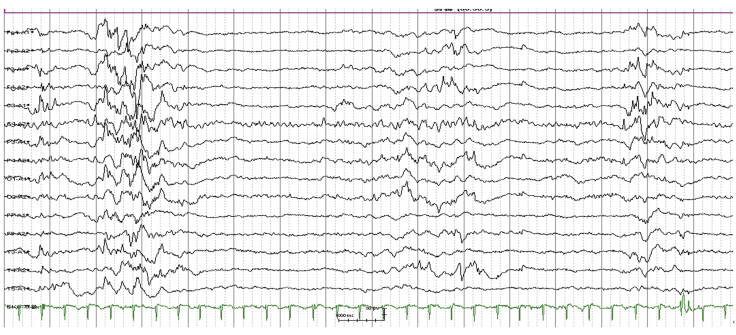
Electroencephalogram: burst–suppression.

As shown in this study, the aEEG tracings in the NKH patient displayed a multitude of abnormalities. A rather featureless background pattern with a lack of normal sleep cycle was noted, which means the child had severe brain damage. 15 Also, the EEG typically showed a burst–suppression pattern. A longer IBI than the corresponding gestational age also suggested that the child might have brain injury. 16 In this study, the EEG findings were similar to aEEG findings. The 24-hour EEG also typically showed a burst–suppression pattern ( Fig. 4 ).

A typical neonatal single seizure was found in Fig. 3A–C , but clinical symptoms were not observed. The presentation of the seizure was subclinical. Because of the paucity of clinical correlates, clinical diagnosis of neonatal seizures is difficult. Early recording of high-risk infants is helpful for the early diagnosis and treatment of seizures. Despite conventional EEG remaining the “gold standard” for epileptic seizures and electroencephalographic assessment of encephalopathic changes, the conventional EEG requires complex equipment and specialized staff to apply the numerous electrodes, and needs neurophysiologists in the clinical centers to interpret the report. 28 Compared with the EEG, the most important thing about aEEG is unnecessary to wait for neurophysiologists to interpret the report. That means the doctors can also detect the epileptic waves and give the appropriate treatment as soon as possible. It is good for the patient. The aEEG examination is more convenient than EEG in clinical settings. For the aEEG, recordings of longer duration can be obtained easily, and the ongoing monitoring of patients is encouraged. However, a review of the raw EEG tracing is indicated to conﬁrm the presence of typical seizure potentials. The aEEG and EEG complement each other.

In previously reported NKH cases, genetic analysis determined that approximately 70 to 80% of the gene mutations were caused by *GLDC* gene mutations, and only 15 to 20% by *AMT* gene mutations. 1 To our knowledge, this is the second case of NKH caused by the *AMT* gene mutation found in Chinese people, and the mutation is homozygous, as follows: homozygous p.(Arg222Cys) mutation. Furthermore, this is the first case report describing the changes in the aEEG pattern of NKH in Chinese people so far. Therefore, it is necessary to report this case.

In conclusion, patients with NKH often exhibit complicated clinical phenotypes and lack specific symptoms, especially the symptoms of encephalopathy are atypical. This case showed that the aEEG monitoring was useful for the early diagnosis and treatment of seizures. It can help the doctor to carry out appropriate treatment as soon as possible. The application value of the aEEG in patients with NKH was significant.
